# The interaction effect of pedagogical agent and emotional feedback on effective learning: a 2 × 2 factorial experiment in online formative assessment

**DOI:** 10.3389/fpsyg.2025.1610550

**Published:** 2025-07-28

**Authors:** Xiaoqin Tang, Li Jiang, Guoli Liu, Hongxia Li

**Affiliations:** ^1^Department of Educational Technology, Sichuan Normal University, Chengdu, China; ^2^Baiyue Chenglong Primary School, Chengdu, China

**Keywords:** online learning, pedagogical agents, emotional feedback, formative assessments, learning performance

## Abstract

**Introduction:**

To address the challenge of face-to-face communication in online learning, integrating pedagogical agents and emotional feedback has been proposed as viable solutions. However, research on their impact during formative assessments remains limited.

**Methods:**

This study therefore conducted a 2 (Pedagogical agent: present vs. absent) × 2 (Emotional feedback: present vs. absent) experimental study using an online learning system to explore their effects on learning performance.

**Results:**

Results indicated that pedagogical agents had a slightly negative influence on transfer scores, while emotional feedback significantly boosted engagement. When both were combined, learners exhibited the highest motivation, although this did not significantly enhance emotional perception or performance and slightly reduced transfer scores. Notably, the use of these tools shortened learning duration.

**Discussion:**

These findings suggest that educators should exercise caution when designing pedagogical agents in online formative assessment environments to avoid potential distractions during the learning process. Meanwhile, the integration of emotional feedback may contribute to creating a more humanized digital learning atmosphere, thereby supporting learners in their online learning experience. Overall, this study provides crucial insights into the complex effects of these tools on learning in computer-based online formative assessments, guiding future design and application.

## 1 Introduction

Feedback is essential for academic development and achievement (Carless, 2019). It provides learners with precise information about the discrepancies between their actual performance and expected standards, guiding them to adjust their learning strategies and behaviors, and ultimately enhancing their educational outcomes (Hattie and Timperley, 2007). Online formative assessment refers to assessments conducted during the online learning process, aiming to provide feedback to facilitate student learning and progress (Kugurakova et al., 2021). Unlike traditional summative assessment, formative assessment focuses more on the learning process, aiming to provide learners with timely feedback through continuous evaluation to enhance their learning and self-regulation abilities (Broadbent et al., 2018). Formative assessment emphasizes timely, concrete, and constructive feedback, as well as deep learner participation and interaction with feedback content (Kugurakova et al., 2021; Gikandi et al., 2011; Nagandla et al., 2018; Sari and Saadah, 2022). However, the distinctive nature of online learning environments, characterized by spatial and temporal separation between learners, instructors, and peers, significantly diminishes the effectiveness of traditional feedback mechanisms (Nicol and Macfarlane-Dick, 2006). Therefore, providing effective feedback to online learners is increasingly a focus of researchers (Krusche and Seitz, 2018; Zheng et al., 2022). Furthermore, while computer-assisted instruction can enhance learning efficiency and personalization, it may also lead to a sense of isolation among learners, depriving them of essential social interaction and learning support (Dinçer and Doğanay, 2017). To address the absence of instructor presence and guidance in online learning environments, pedagogical agents have been developed. Pedagogical agents are computer-based characters or avatars designed to simulate human teachers in order to deliver educational content and provide guidance (Zhao et al., 2023). Pedagogical agents, as virtual avatars, can simulate human roles, offering new avenues for emotional communication, learning guidance, and social interaction (Tao et al., 2022). Numerous studies have indicated that using pedagogical agents to provide immediate feedback to online learners can stimulate their interest in learning, thereby improving their learning experience and performance (Wang et al., 2022; Schneider et al., 2022). Studies also confirm that pedagogical agents with emotional support and feedback (such as encouragement and praise) can create a positive learning environment, enhance learners’ intrinsic motivation and engagement, and improve their learning experience (Wang Y. et al., 2023; Lang et al., 2022).

While numerous studies have explored the role of pedagogical agents and their feedback in computer-based environments, there are still inconsistencies and unexplored areas in the current research. Previous research on agent feedback has primarily focused on single learning scenarios such as video instruction, with most studies employing discrete feedback events (Lawson et al., 2021; Schneider et al., 2022; Jing et al., 2022; Wang Y. et al., 2023). These feedback mechanisms struggle to meet the dual needs of cognitive and emotional support for learners during the learning process. Online formative assessment, as an important tool for online learning support, emphasizes the characteristics of immediate feedback and deep participation of students (Gikandi et al., 2011). Therefore, when learners participate in the online formative assessment process, will the presence of emotional feedback and teaching agents play the expected roles? Will these two factors have an interactive impact on students’ effective participation? Therefore, this study aims to investigate the impact of different feedback forms in online formative assessments on learners’ learning outcomes (motivation, experience, and achievement) through a 2 (Pedagogical agents: present vs. absent) × 2 (Emotional feedback: present vs. absent) experimental study. The study seeks to provide a scientific basis and effective strategies for optimizing feedback in online formative assessments and enhancing online learning effectiveness.

## 2 Literature view and research hypothesis

### 2.1 The impact of emotional feedback on online learning

The use of computer technology to provide feedback to learners in online learning environments is considered as an important way to enhance learning outcomes (Pardo et al., 2019). However, Förster et al. (2018) clearly noted that the degree of feedback’s impact on learning performance can vary significantly. While some researchers argue that feedback enhances learning performance (Lim et al., 2021), contrasting findings from other studies suggest that feedback may not exert a significant impact on learning performance (Janelli and Lipnevich, 2021; Sun and Yeh, 2017). The significance of feedback quality has been acknowledged by numerous researchers (Jensen et al., 2023; Van der Meij et al., 2015; Wang et al., 2019). Economides (2005) categorized instructional feedback into cognitive and emotional feedback. Cognitive feedback provides learners with information related to cognition, aiding them in understanding and solving problems, while emotional feedback aims to enhance learners’ emotional states. In recent years, researchers have increasingly recognized the pivotal role of learners’ emotional states in multimedia learning environments, paralleling the importance of cognitive factors (Lang et al., 2024). The Cognitive Affective Theory of Learning with Media (CATLM) highlights the significant impact of emotional interaction between learners and computers on cognitive processing of multimedia information (Moreno and Mayer, 2007). Building on this, the Integrated Cognitive Affective Model of Learning with Multimedia (ICALM) emphasizes the inseparable nature of emotions and cognitive processes. Instructional design with emotional support can positively affect learners’ emotional experiences, cognitive processing, and academic performance (Plass and Kaplan, 2016). Furthermore, when learners receive scores below their expectations on a learning task, they are more likely to experience negative emotions as a result of the feedback (Ryan and Henderson, 2018). Thus, feedback should address both cognitive and emotional needs, necessitating online learning systems to provide warm and humane emotional feedback.

Karunarathne et al. (2024) found that distance learners expect feedback that is emotionally supportive. A similar qualitative study found that learners generally believe effective feedback should be emotionally supportive (Dawson et al., 2019). Given that emotional feedback is more likely to affect learners through a combination of cognitive and emotional pathways, existing research has begun to focus on process variables such as learners’ emotional perception and learning experience, which can better capture its comprehensive impact on the entire learning process, especially in the context of formative assessment. In the realm of online formative assessment, research on Intelligent Tutoring Systems (ITS) has indicated that emotional feedback may influence the learning process. For instance, Jiménez et al. (2018) discovered that incorporating positive emotional feedback within ITS can enhance learning motivation and improve the overall learning experience. Similarly, Liu et al. (2022) discovered that adaptive learning systems based on emotional feedback significantly improved learning efficiency and reduced learning anxiety. However, the impact of emotional feedback on learner outcomes is not consistent, with numerous studies finding no significant effect of emotional feedback on academic performance (Kim et al., 2017; Terzidou et al., 2018). This contradiction may stem from differences in feedback media, particularly the limitations of single-text feedback formats. Existing research has proven that multimodal feedback is more conducive to learning than pure text feedback (Swart et al., 2019). In light of this, the present study constructs a synchronous feedback mechanism of text and voice in the online formative assessment system to deliver richer audiovisual stimuli. We aim to more comprehensively evaluate the potential of emotional feedback in enhancing learning experience, boosting motivation, and promoting academic achievement in online formative assessment by integrating dual audiovisual stimuli.

### 2.2 The impact of pedagogical agents on online learning

Pedagogical agents are virtual characters designed to provide educational services and immediate feedback in online learning, fulfilling the social role of teachers (Yilmaz and Karaoglan Yilmaz, 2020). Previous research has examined the benefits of pedagogical agents for learners from both theoretical and empirical perspectives, yet many debates remain. Theoretically, Social Presence Theory and Social Agency Theory posit that pedagogical agents can evoke positive emotions in learners, enhancing their satisfaction and learning outcomes (Gunawardena and Zittle, 1997; McLaren et al., 2011). On the other hand, Interference Theory and Cognitive Load Theory suggest that the presence of pedagogical agents can be a burden to learners (Moreno et al., 2001; Sweller et al., 2019). Both theories assert that individuals have limited memory capacity, and pedagogical agents, being irrelevant to the learning task, can impose additional cognitive load on learners. Thus, cognitive load has become a crucial variable in examining whether pedagogical agents cause learning interference. Moreover, the anthropomorphic features of pedagogical agents—such as visual cues and human-like conversational styles—may influence learners’ social presence during the learning process as a form of social cue, thereby further affecting their emotions, engagement, and motivation levels (Sun et al., 2024; Zhang et al., 2024; Wang Y. et al., 2023). Other researchers have empirically investigated the impact of pedagogical agents on learner outcomes. For instance, research by Schneider et al. (2022) confirms that pedagogical agents can activate positive emotions in learners, leading to a better learning experience and improved academic performance. A recent meta-analysis also indicates that the implementation of pedagogical agents can enhance learning outcomes (Castro-Alonso et al., 2021). However, some studies show that pedagogical agents have no significant impact or even a negative effect on learning outcomes (Lin et al., 2013; Liew et al., 2013).

Moreover, the role of pedagogical agents in online formative assessment environments remains unclear. Formative assessment environments are characterized by their process-oriented and continuous nature, aiming primarily to support learners’ development throughout the learning process (Parmigiani et al., 2024). This contrasts significantly with typical instructional video scenarios. Thus, although most studies have confirmed the positive impact of pedagogical agents on learning, whether they can also facilitate learning in the context of formative assessment remains an incompletely understood question.

### 2.3 The interactive effects of pedagogical agents and emotional feedback on online learning

Research on the interactive effects of pedagogical agents and emotional feedback is still in the exploratory stage. Some studies have shown a synergistic effect between the presentation of pedagogical agents (e.g., anthropomorphic appearance) and emotional feedback (e.g., empathetic expression). For example, Horovitz and Mayer (2021) found that agents with emotional feedback can effectively enhance learners’ motivation. A meta-analysis also revealed that the combined use of pedagogical agents and emotional feedback can elicit positive emotions, enhance intrinsic motivation, and thereby facilitate learning (Wang Y. et al., 2023). Lang et al. (2024) further uncovered the dynamic effects of this synergistic interaction. They found that, compared to non-supportive agents, the combination of pedagogical agents and emotional feedback can reduce learners’ frustration, improve emotional experience, and guide learners to adopt more effective learning strategies. This indicates that the synergy between pedagogical agents and emotional support not only affects learners’ emotions and motivation in the short term but also influences the selection of learning strategies through dynamic interaction. To capture this complex mechanism of action, this study integrates multiple dependent variables, starting from two cognitive dimensions: learning performance and cognitive load, while also incorporating key non-cognitive indicators such as learning motivation, academic emotions, learning engagement, and social presence to present the dynamic impact of the interaction between pedagogical agents and emotional feedback on the entire learning process. However, some studies have found that the combination of agents and emotional feedback does not yield better learning outcomes (Wang et al., 2022). Moreover, Ba et al. (2021) combined the embodiment, voice, and emotional cues of agents to convey emotional signals through both visual and auditory channels. The results showed that, compared to neutral or non-embodied agents, learners had better knowledge transfer scores under conditions with embodied agents that conveyed emotional cues. Although many studies have demonstrated the benefits of combining pedagogical agents with emotional feedback for learning, the underlying synergistic mechanisms remain unclear. Therefore, further research is needed to explore the interaction between agents and emotional feedback in the context of online formative assessment environments.

In summary, this study investigates the impact of feedback agents and emotional feedback on college students’ learning performance within the online formative assessment. Learning performance encompass both cognitive aspects (knowledge retention, knowledge transfer, cognitive load) and non-cognitive aspects (study duration, learning motivation, academic emotions, social presence, and learning engagement). The specific research questions and hypotheses are as follows:

*RQ1*: How does the presence of pedagogical agents affect the learning performance within the online formative assessment?

*H1a*: The use of pedagogical agents will lead to fewer negative emotions, higher positive emotions, learning motivation, social presence, and learning engagement, as well as longer study duration compared to not using pedagogical agents.

*H1b*: The use of pedagogical agents increases learners’ extraneous cognitive load and intrinsic cognitive load, diverting more of their attention.

*RQ2*: How does the presence of emotional feedback affect the learning performance within the online formative assessment?

*H2a*: Learners who receive emotional feedback will experience fewer negative emotions, more positive emotions, learning motivation, social presence, and learning engagement, as well as longer study duration compared to learners who do not receive emotional feedback.

*H2b*: Compared to conditions without emotional feedback, emotional feedback aids in learners’ better academic performance and reduces their cognitive load, particularly the extraneous cognitive load.

*RQ3*: How do pedagogical agents and emotional feedback interact to affect the learning performance within the online formative assessment?

*H3*: Online learners will demonstrate better learning experiences and performance under conditions where pedagogical agents and emotional feedback are combined.

## 3 Method

### 3.1 Research design

**Figure 1:**
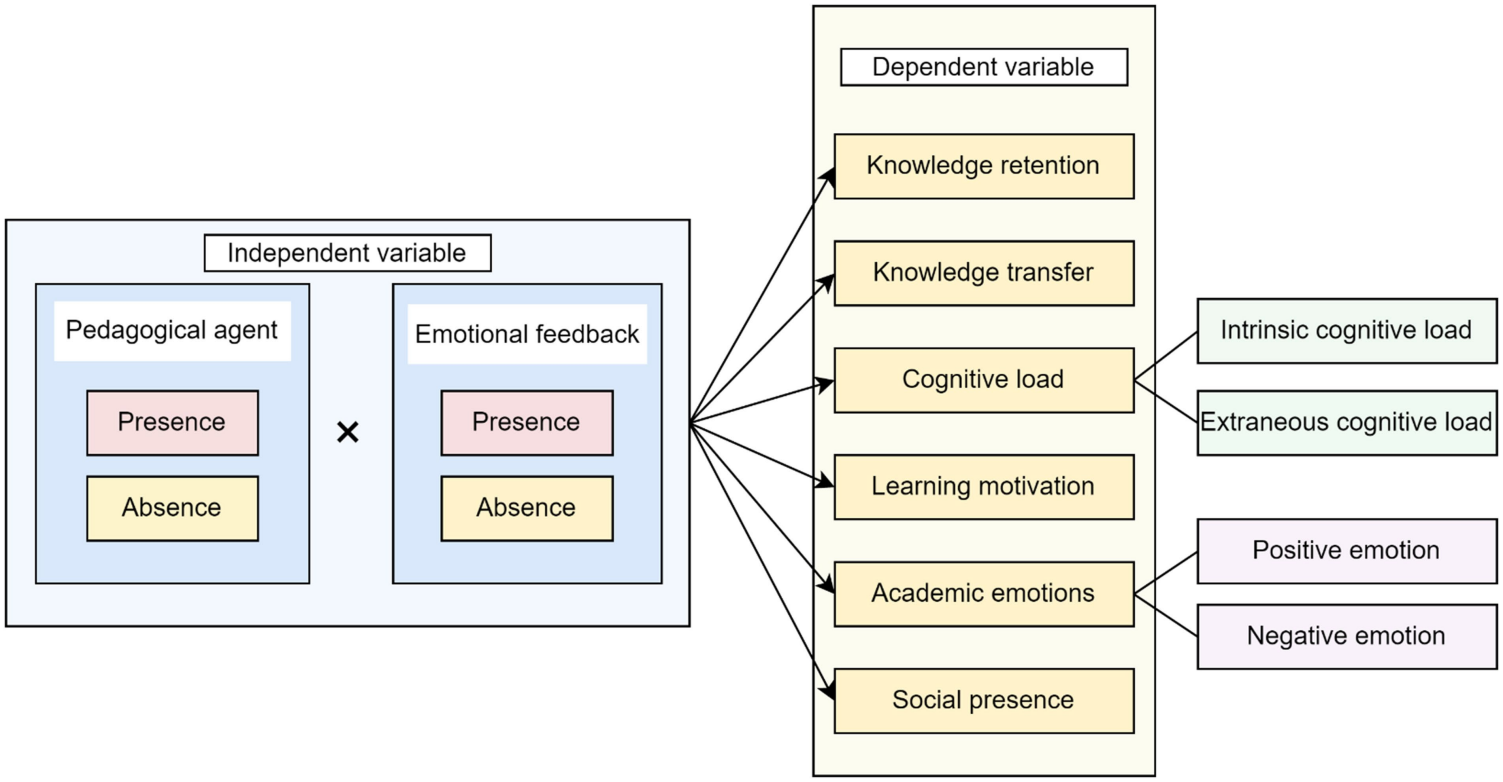
Research design.

This study employs a 2 (Pedagogical agents: present vs. absent) × 2 (Emotional feedback: present vs. absent) between-subjects experimental design. The research variables are depicted in Figure 1.

**Figure 2:**
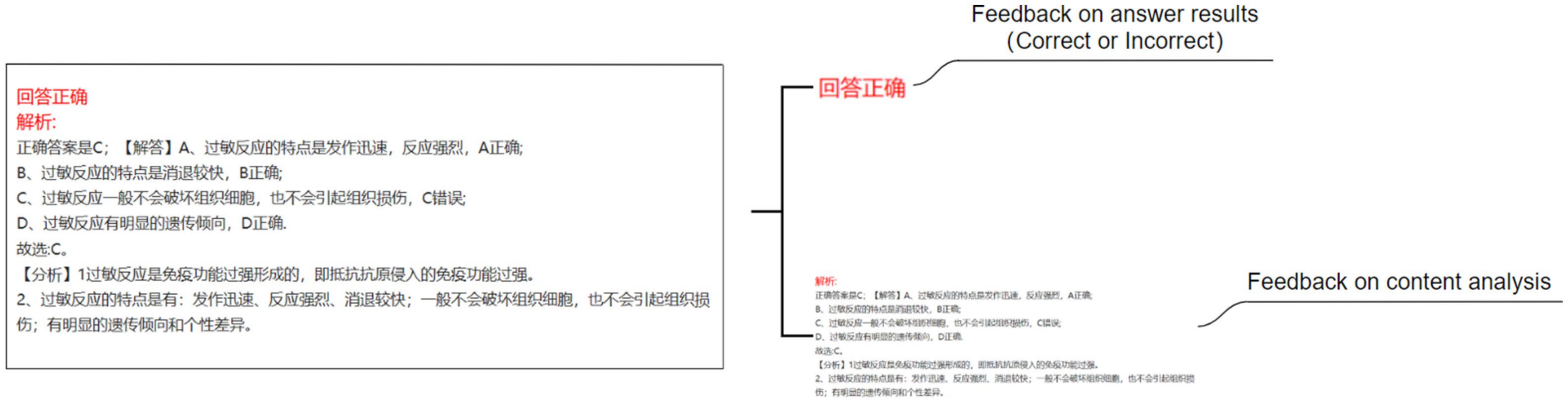
No pedagogical agent and no emotional feedback (NN).

**Figure 3:**
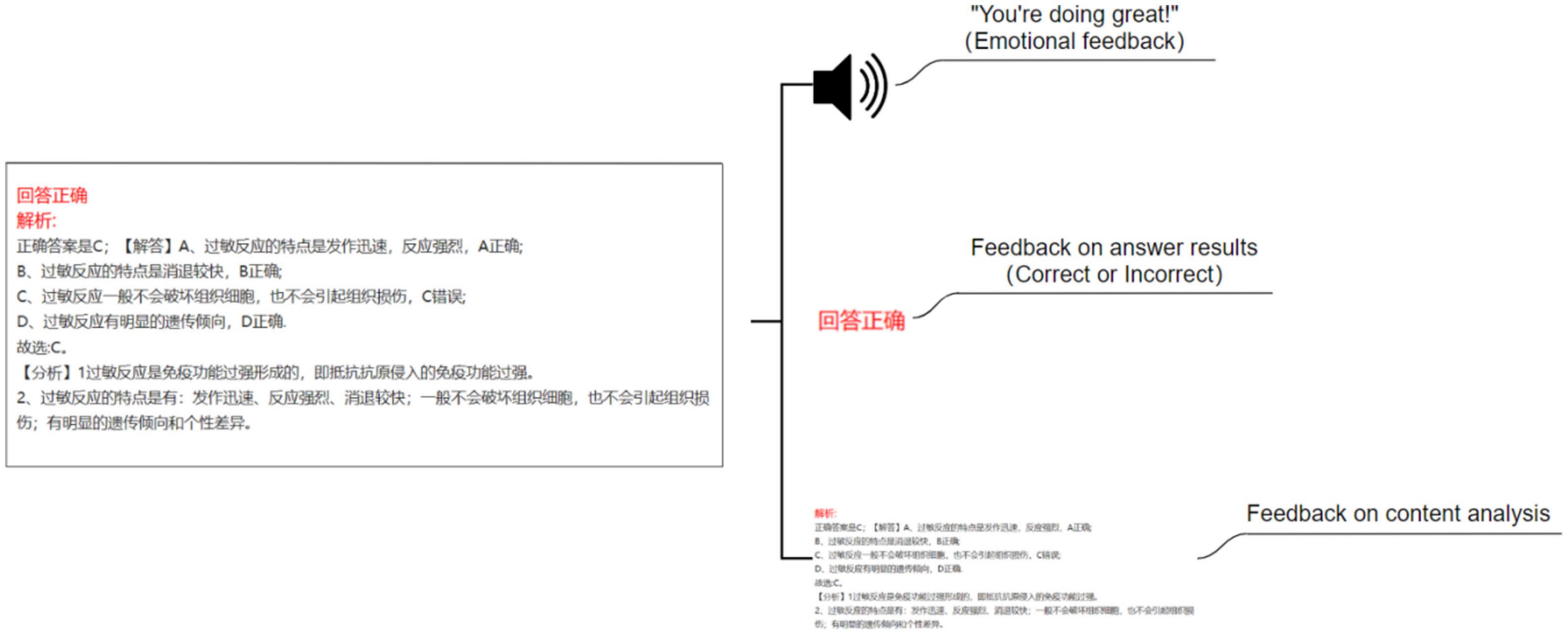
No pedagogical agent but with emotional feedback (NE).

**Figure 4:**
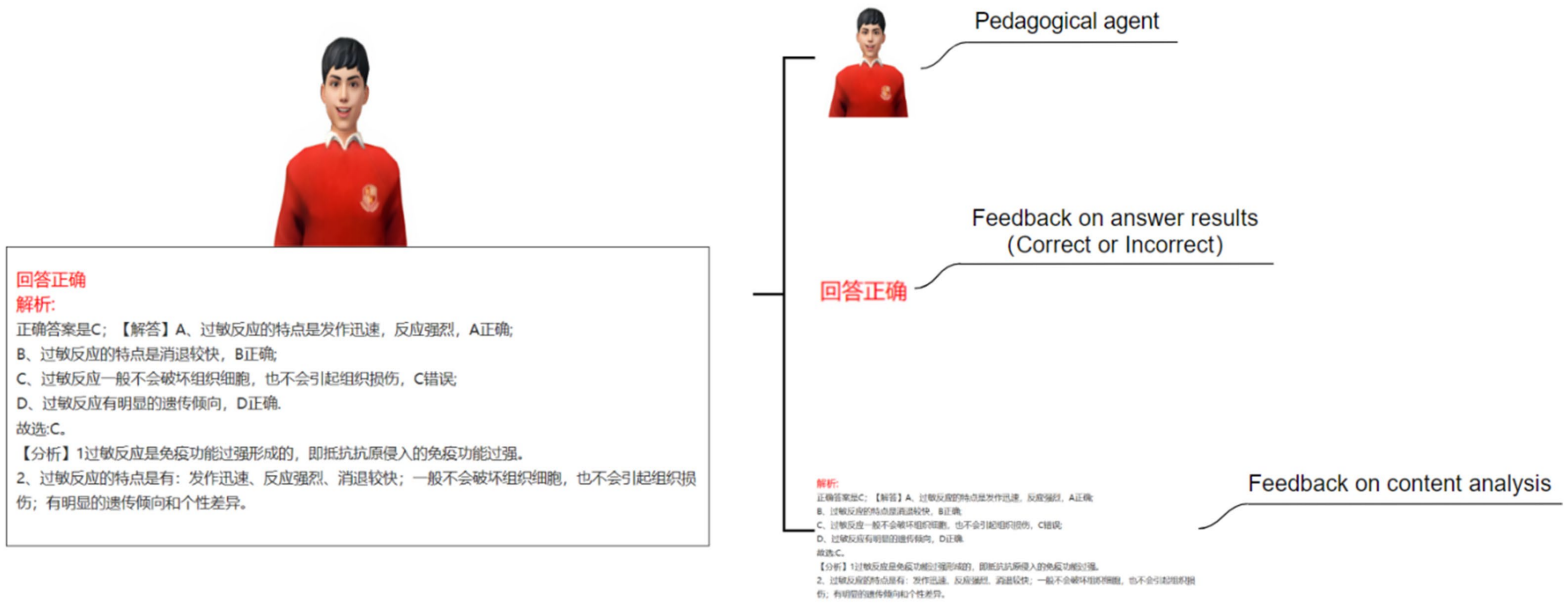
With a pedagogical agent but without emotional feedback (AN).

**Figure 5:**
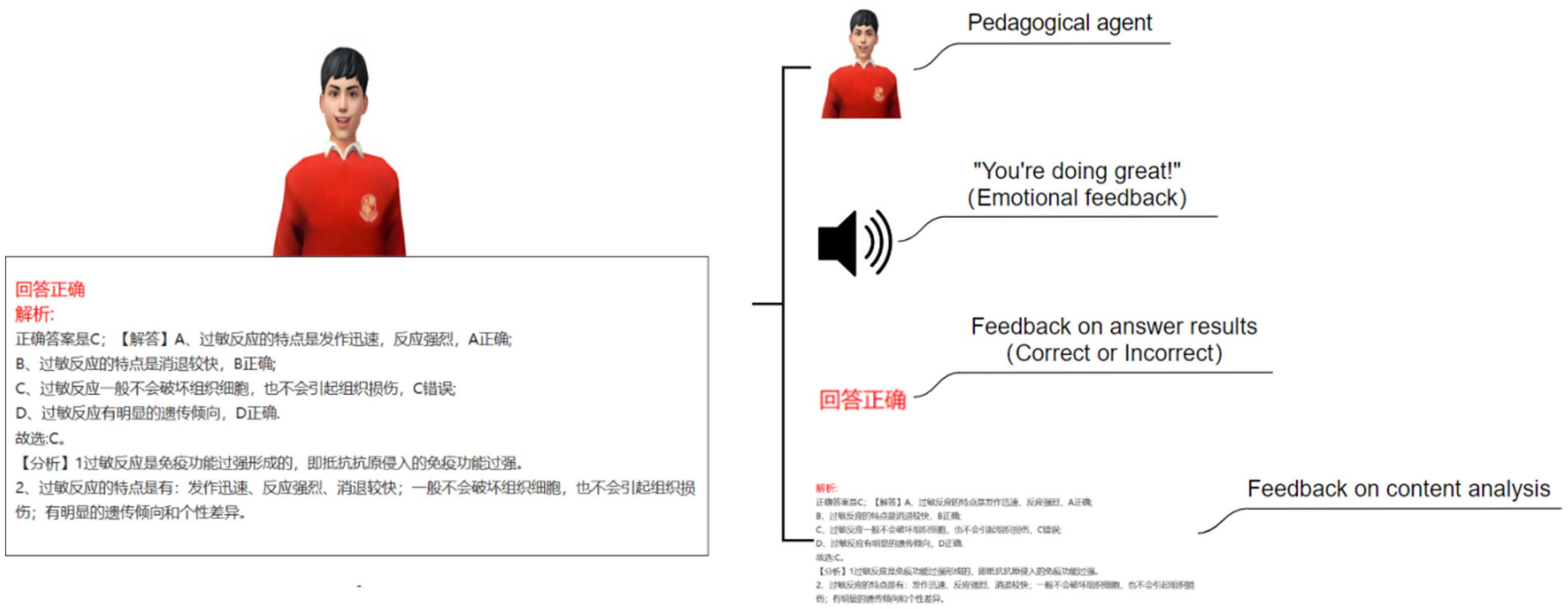
With both a pedagogical agent and emotional feedback (AE).

**Figure 6:**
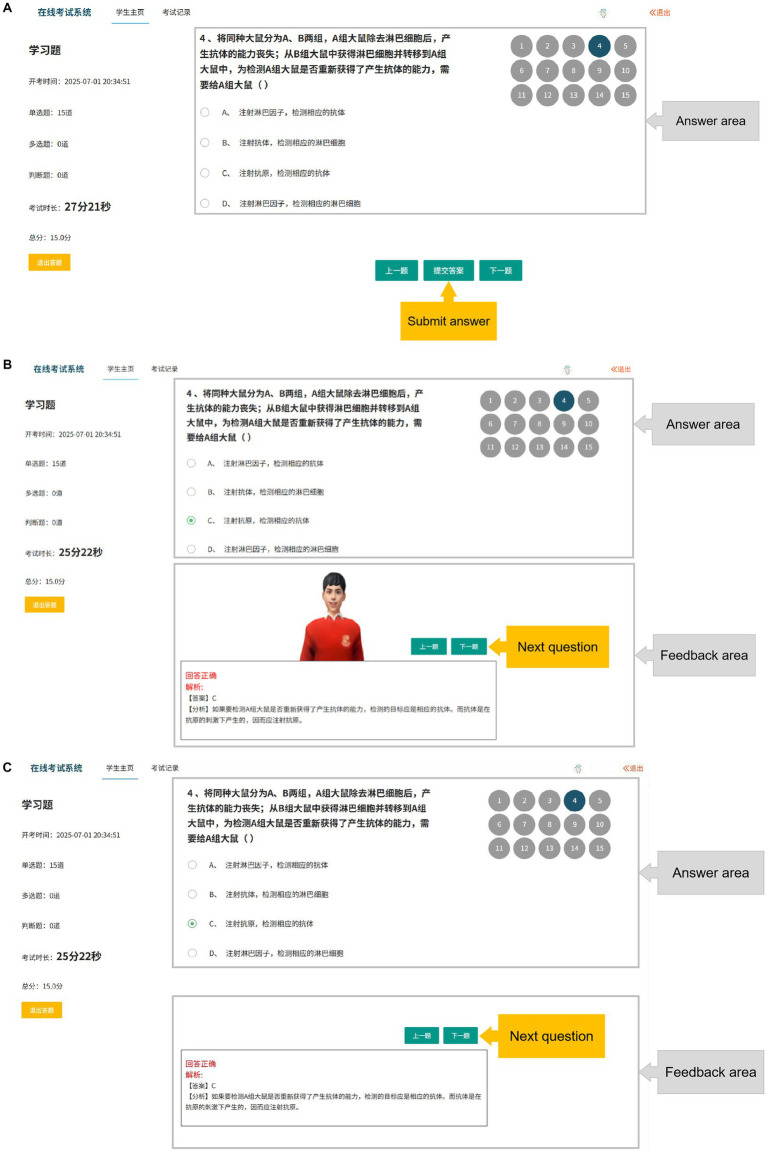
Schematic diagram of the learning system interface: **(A)** Screenshot of the learning system interface before learners submit their answers (Answer area: Where learners provide their responses. Submit answer: Learners click this option to submit their answers after making a choice) **(B)** Screenshot of the learning system interface with a pedagogical agent after learners submit their answers (Answer area: Where learners provide their responses. Feedback area: Where the system provides feedback from the pedagogical agent and cognitive feedback. Next question: Learners can click this option to move on to the next question after viewing the feedback) **(C)** Screenshot of the learning system interface without a pedagogical agent after learners submit their answers (Answer area: Where learners provide their responses. Feedback area: Where the system provides feedback from the pedagogical agent and cognitive feedback. Next question: Learners can click this option to move on to the next question after viewing the feedback).

This study established four experimental conditions: no feedback agent and no emotional feedback (NN) (see Figure 2), no pedagogical agent but with emotional feedback (NE) (see Figure 3), with a pedagogical agent but without emotional feedback (AN) (see Figure 4), and with both a pedagogical agent and emotional feedback (AE) (see Figure 5). For each condition, a computer feedback system was independently developed, named Learning System I, Learning System II, Learning System III, and Learning System IV, corresponding to the aforementioned experimental conditions. The learning content in the four learning systems are identical, derived from high school biology knowledge related to immune system regulation, consisting of a total of 15 multiple-choice questions. For example: “In patients who have recovered from COVID-19 after treatment, what is most likely to persist long-term in the body? (A) Memory cells. (B) COVID-19 virus. (C) Antibodies. (D) Plasma cells.” As learners use the learning system, the system automatically records the duration that learners in each group spend viewing explanations about the answer (feedback). Specifically, when a learner clicks on “Submit answer” (see Figure 6A), the system automatically provides the question analysis and starts timing. The timing stops when the learner clicks on “Next question” (see Figures 6B,C). Ultimately, the learning system aggregates the explanation viewing durations for all questions, which we use as each learner’s study duration. This measure aims to reflect the extent of learners’ attention to feedback information and the depth of their cognitive processing. It also captures at the behavioral level whether the interventions of pedagogical agents and emotional feedback truly prompt learners to utilize the feedback content for learning. In the learning system with a pedagogical agent, when learners view the explanation after completing a question, the explanation and the pedagogical agent appear simultaneously (see Figure 6B). The pedagogical agent is specifically an image of a man from the shoulders up, capable of providing learners with either emotional or non-emotional feedback in the form of voice, accompanied by slight head movements, lip-synced narration, and eye contact. In the condition without an agent, learners see only the content of the question explanation (see Figure 6C). In terms of emotional feedback, vocal praise is given when the subject answers correctly (e. g., “You’re doing great!”), and neutral encouragement is offered when the answer is incorrect (e. g., “Keep going, do not lose heart.”). Such vocal feedback is not provided in the no emotional feedback group.

### 3.2 Participants

This study utilized a 2 × 2 between-subjects factorial design. A total of 112 university students were randomly recruited from a specific university to participate in the experiment. Participants were randomly assigned to four experimental conditions, with detailed information presented in Table 1. Drawing on the research design and sample sizes of comparable studies (Beege et al., 2020; Van Yperen, 2003), we believe that the sample size of our study is sufficient for fundamental statistical analysis. All data and responses of the students were handled anonymously. As per the university’s ethics committee guidelines, this project falls under the category of evaluation work and is not subject to further approval.

**Table 1:** Descriptive statistics of participants in different learning systems (experimental conditions).

Group	Number of participants (*n*)	Gender [(%)]		Age	
		Male	Female	*M*	SD
Learning System I (NN)	27	9 (33.3)	18 (66.7)	19.85	1.26
Learning System II (NE)	26	12 (46.2)	14 (53.8)	20.58	2.10
Learning System III (AN)	28	5 (17.9)	23 (82.1)	19.43	0.96
Learning System IV (AE)	31	14 (45.2)	17 (54.8)	20.45	1.12

### 3.3 Measures

#### 3.3.1 Pre-test

The pre-test consists of 10 items, including 9 multiple-choice questions (each worth 2 points) and 1 true/false question (also worth 2 points), with a total possible score of 20 points. Both multiple-choice and true/false questions pertain to fundamental knowledge of human immunity. An example question is “Some people develop allergic rhinitis when they inhale allergens like pollen.” The correct understanding of allergies is (). (A) Allergies are a normal reaction to ‘non-self’ substances. (B) Allergic symptoms appear upon first contact with an allergen. (C) Allergies exhibit significant individual differences and genetic tendencies. (D) Antibodies bind to allergens and then adhere to mast cells. All questions in the pre-test were of an objective nature, derived from established test items to ensure consistency and validity in scoring.

#### 3.3.2 Learning performance

Learning performance encompasses the post-test and knowledge transfer test, which are used to assess the immediate memory effect and transfer ability of learners after using the learning system. The study administered the post-test and knowledge transfer test to the learners shortly after they completed the learning (about 10 min, during which they completed the subjective questionnaire). The post-test consists of 7 questions, including 6 multiple-choice questions and 1 fill-in-the-blank question with three answer spaces. Each multiple-choice question is worth 2 points, and each blank space in the fill-in-the-blank question is worth 2 points, with a total possible score of 18 points. An example question is “The immune system consists of (), (), and () three parts.” All questions in the post-test were of an objective nature, derived from established test items to ensure consistency and validity in scoring. The knowledge transfer test includes 3 short-answer questions, with each question worth 10 points, for a total possible score of 30 points. We have established specific scoring points for each question, and the students’ final scores are determined based on the correct scoring points they have answered. An example question is “When a patient is initially infected with the Dengue virus, G antibodies can be detected in the body after 14 days. Upon re-infection, antibodies can be detected the next day. Please briefly explain the reason for the rapid appearance of G antibodies upon re-infection.” All questions were derived from established test items to ensure validity. The knowledge transfer scores were independently rated by two well-trained raters (*r* = 0.97), and the mean of their ratings was used as the final score.

#### 3.3.3 Study duration

After a learner completes a question and clicks “Submit Answer,” the system automatically provides the solution. If the system includes a pedagogical agent or emotional feedback, these appear simultaneously with the solution. The system then records the duration for which students view the solution, stopping when they select the “next question” option. This viewing duration is recorded for each question and accumulated across all questions. The total study duration is measured by the cumulative time spent viewing solutions, recorded in seconds.

#### 3.3.4 Learning motivation

In this study, a scale developed by Stull et al. (2018) was selected to assess participants’ learning motivation. The scale consists of 6 items using a 5-point Likert scale, where 1 indicates ‘strongly disagree’ and 5 indicates ‘strongly agree’. Higher scores indicate a stronger learning motivation among participants. The questionnaire has a high reliability level, with a Cronbach’s *α* of 0.923. An example item is “I would be interested in learning more about the content presented in this session.”

#### 3.3.5 Cognitive Load

Based on the work of Klepsch et al. (2017), the measure includes two sub-dimensions: intrinsic cognitive load and extraneous cognitive load. The intrinsic cognitive load consists of a single item (“For mastering this topic, many pieces of knowledge need to be kept in mind simultaneously.”) The extraneous cognitive load dimension also consists of a single item (“I think the design of this task was very inconvenient for learning.”) The scale uses a 5-point Likert scale for scoring, where 1 indicates ‘strongly disagree’ and 5 indicates ‘strongly agree’.

#### 3.3.6 Academic emotions

The questionnaire, adapted from Watson et al. (1988), measures students’ academic emotions on two dimensions: positive and negative. Each dimension is assessed with four items, scored on a 5-point Likert scale. Examples are “I feel excited” (positive) and “I feel bored” (negative). Both scales show satisfactory internal consistency reliability (Cronbach’s *α* = 0.887 for positive, 0.865 for negative).

#### 3.3.7 Social presence

The questionnaire was adapted from Law et al. (2019) and contains 3 question items, all on a 5-point Likert scale. An example item is “I enjoy learning in this kind of environment. “The scale has good reliability (Cronbach’s *α* = 0.818).

#### 3.3.8 Learning engagement

This questionnaire is adapted from Paas (1992) and utilizes a 9-point symmetric rating scale. It asks learners to quantify the mental effort or engagement they perceive while completing tasks. The scale ranges from 1 to 9, with 1 indicating very low and 9 indicating very high levels of perceived effort or engagement.

### 3.4 Procedure

**Figure 7:**
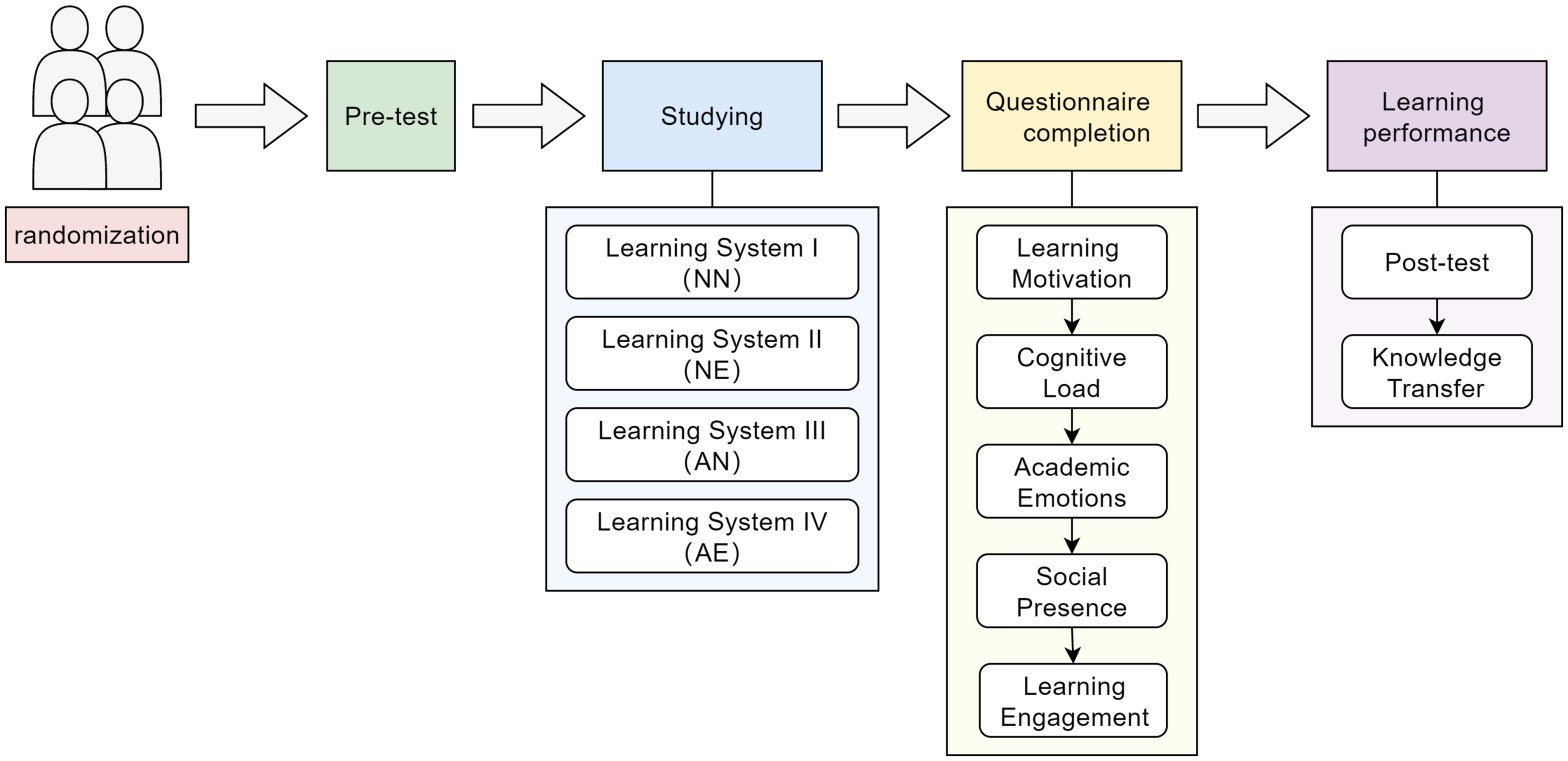
Experimental procedure.

The entire experimental procedure is divided into five steps (as shown in Figure 7). Step 1: Learners are randomly assigned to one of the four experimental conditions and receive an introduction and explanation of the experiment. Step 2: Learners complete a paper-based pre-test. Step 3: Following the pre-test, students use the online learning system, while wearing wired headphones, to register and study. The learning system automatically records the duration for which learners view feedback. Step 4: Students complete an online learning experience questionnaire, which primarily includes items about learning motivation, cognitive load, academic emotions, social presence, and learning engagement. Step 5: Students complete a paper-based post-test. Upon completion of the entire experiment, students receive a small gift as a token of appreciation.

### 3.5 Data analysis

To investigate the impact of pedagogical agents and emotional feedback on students, we conducted a series of 2 (Pedagogical agent: present vs. absent) × 2 (Emotional feedback: present vs. absent) analysis of covariance (ANCOVA) on students’ academic performance, study duration, learning motivation, cognitive load, academic emotions, social presence, and learning engagement, with age as a covariate. Throughout the study, IBM SPSS Statistics 24.0 was utilized for data analysis and statistical testing of all data collected from the experiment, while RStudio was employed for the visualization of the results.

## 4 Research results

This study employed analysis of variance (ANOVA) to examine the differences in pre-test scores, learning system scores, and age across four experimental conditions, and Chi-square tests were used to analyze the differences in gender composition. The results indicated that there were no significant differences in pretest scores (*F* = 0.323, *p* = 0.809), learning system scores (*F* = 0.472, *p* = 0.702), or gender (*χ*^2^ = 6.395, *p* = 0.094) across groups, but there was a significant difference in age (*F* = 4.030, *p* = 0.009). Consequently, age was controlled for as a covariate in subsequent analyses, and an ANCOVA was used to explore the effects of agents and emotional feedback on learning performance. This study further analyzed the experimental results using violin plots and box plots. The descriptive results for all variables are shown in Table 2.

**Table 2:** Ms and SDs of all variables in experiment.

Dependent variable	Experiment condition			
	NN (*M ± SD*)	NE (*M ± SD*)	AN (*M ± SD*)	AE (*M ± SD*)
Knowledge retention	9.48 *±* 3.70	10.23 *±* 2.96	10.07 *±* 4.44	9.10 *±* 2.87
Knowledge transfer	15.37 *±* 3.47	11.54 *±* 6.09	11.14 *±* 8.29	11.42 *±* 5.48
Study duration	443.00 *±* 360.13	188.27 *±* 114.51	159.11 *±* 115.96	165.97 *±* 121.96
Learning motivation	3.61 *±* 0.73	3.30 *±* 0.90	3.43 *±* 0.68	3.75 *±* 0.87
Extraneous cognitive load	2.75 *±* 0.65	2.58 *±* 0.81	3.04 *±* 0.64	2.84 *±* 0.79
Intrinsic cognitive load	2.93 *±* 0.83	3.08 *±* 0.98	3.36 *±* 0.95	3.10 *±* 0.79
Positive emotion	3.56 *±* 0.63	3.63 *±* 0.72	3.42 *±* 0.84	3.67 *±* 0.58
Negative emotion	2.45 *±* 0.64	2.38 *±* 1.04	2.59 *±* 0.80	2.44 *±* 0.62
Social presence	3.42 *±* 0.62	3.62 *±* 0.72	3.46 *±* 0.57	3.64 *±* 0.62
Learning engagement	4.96 *±* 0.98	5.62 *±* 0.98	5.29 *±* 1.01	5.61 *±* 0.88

### 4.1 Learning performance

In terms of post-test, neither the pedagogical agent [*F*(1, 108) = 0.331, *p* = 0.566, *η*^2^ = 0.003] nor the emotional feedback [*F*(1, 108) = 0.124, *p* = 0.726, *η*^2^ = 0.001] had a significant main effect, and their interaction was also not significant [*F*(1, 108) = 1.448, *p* = 0.232, *η*^2^ = 0.013].

**Figure 8:**
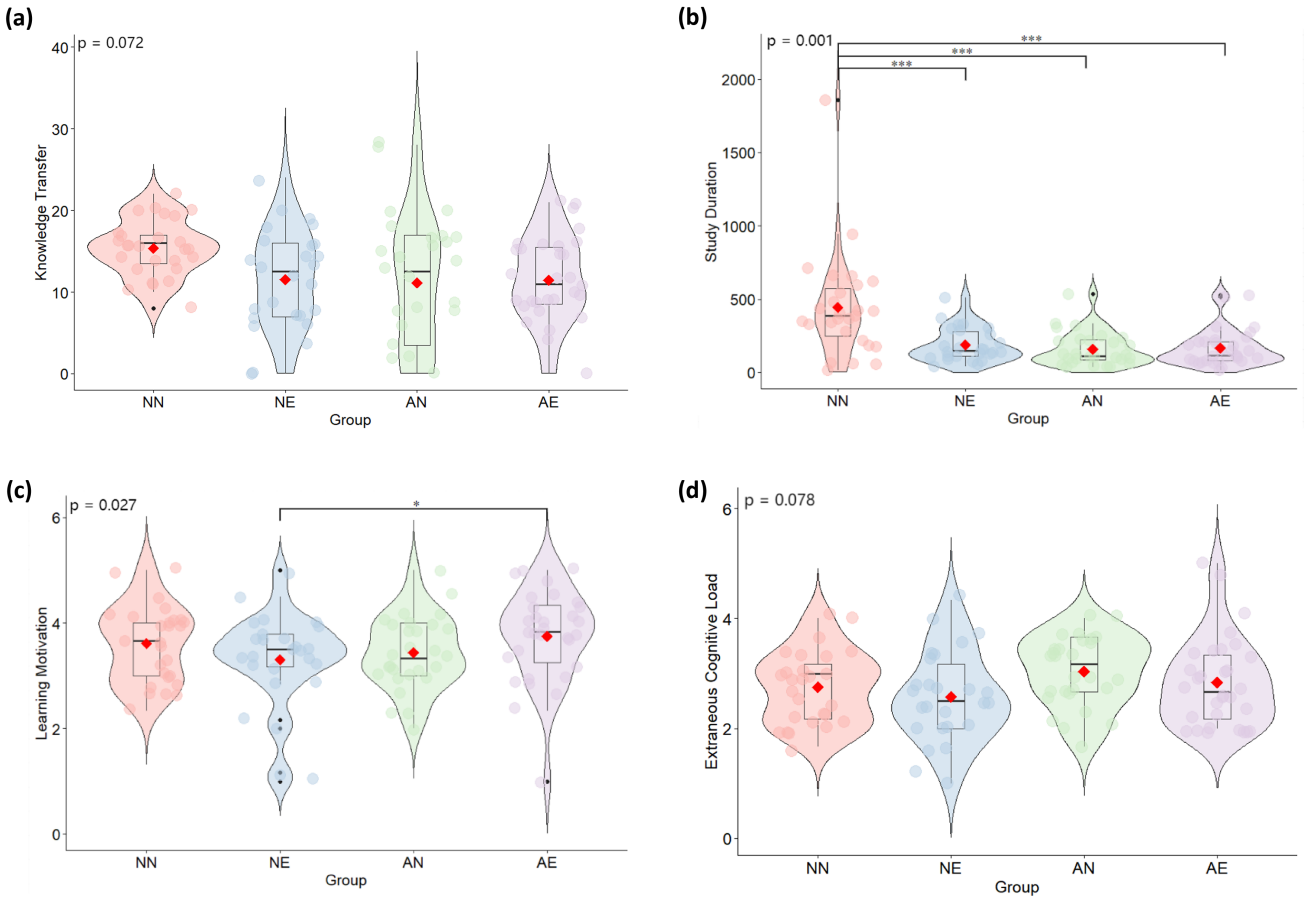
Performance of selected variables under different conditions: **(a)** Knowledge transfer. **(b)** Study duration. **(c)** Learning motivation. **(d)** Extraneous cognitive load. ***p < 0.001, **p < 0.01, *p < 0.05.

In the domain of knowledge transfer, the pedagogical agent showed a marginally significant main effect [*F*(1, 108) = 3.809, *p* = 0.054, *η*^2^ = 0.034], whereas the main effect of emotional feedback was not significant [*F*(1, 108) = 1.519, *p* = 0.220, *η*^2^ = 0.014]. The interaction between the two was also marginally significant [*F*(1, 108) = 3.331, *p* = 0.072, *η*^2^ = 0.030]. To better understand this result, we conducted simple effects analysis on the means obtained under different experimental conditions, as shown in Figure 8a. The results revealed that when the pedagogical agent was absent, learners in the emotional feedback condition had significantly lower knowledge transfer than those without emotional feedback (*F* = 4.364, *p* = 0.039, *η*^2^ = 0.040). When the pedagogical agent was present, there was no significant difference between the emotional feedback and no emotional feedback conditions (*F* = 0.137, *p* = 0.712, *η*^2^ = 0.001). When emotional feedback was absent, learners with the pedagogical agent had significantly lower knowledge transfer than those without the pedagogical agent (*F* = 6.749, *p* = 0.011, *η*^2^ = 0.060). However, when emotional feedback was present, there was no significant difference between the pedagogical agent and no pedagogical agent conditions (*F* = 0.010, *p* = 0.922, *η*^2^ = 0.000). This suggests that using either the pedagogical agent or emotional feedback alone may hinder knowledge transfer, while using both together may offset their respective negative effects to some extent.

### 4.2 Study duration

The pedagogical agent [*F*(1, 108) = 15.237, *p* = 0.000, *η*^2^ = 0.125] and emotional feedback [*F*(1, 108) = 9.524, *p* = 0.003, *η*^2^ = 0.082] both had significant main effects on the duration learners spent reviewing feedback. Additionally, the interaction between the pedagogical agent and emotional feedback was significant [*F*(1, 108) = 11.231, *p* = 0.001, *η*^2^ = 0.095]. Further simple effects analysis was conducted, as shown in Figure 8b. When the pedagogical agent was absent, the study duration in the emotional feedback condition was significantly lower than that in the no emotional feedback condition (*F* = 24.950, *p* = 0.000, *η*^2^ = 0.191). However, when the pedagogical agent was present, there was no significant difference between the emotional feedback and no emotional feedback conditions (*F* = 0.046, *p* = 0.830, *η*^2^ = 0.000). When emotional feedback was absent, the study duration with the pedagogical agent was significantly lower than that without the pedagogical agent (*F* = 29.136, *p* = 0.000, *η*^2^ = 0.216). When emotional feedback was present, there was no significant difference between the pedagogical agent and no pedagogical agent conditions (*F* = 0.262, *p* = 0.610, *η*^2^ = 0.002). These results indicate that the presence of either the pedagogical agent or emotional feedback alone can significantly reduce the study duration of learners.

### 4.3 Learning motivation

The main effects of the pedagogical agent [*F*(1, 108) = 0.464, *p* = 0.497, *η*^2^ = 0.004] and emotional feedback [*F*(1, 108) = 0.570, *p* = 0.452, *η*^2^ = 0.005] on learning motivation were both non-significant, but the interaction between them was significant [*F*(1, 108) = 5.011, *p* = 0.027, *η*^2^ = 0.045]. Further simple effects analysis was conducted, as shown in Figure 8c. When the pedagogical agent was absent, there was no significant difference between the emotional feedback and no emotional feedback conditions (*F* = 1.220, *p* = 0.272, *η*^2^ = 0.011). However, when the pedagogical agent was present, learning motivation was significantly higher in the emotional feedback condition than in the no emotional feedback condition (*F* = 4.696, *p* = 0.032, *η*^2^ = 0.042). When emotional feedback was absent, there was no significant difference between the pedagogical agent and no pedagogical agent conditions (*F* = 1.552, *p* = 0.216, *η*^2^ = 0.014). When emotional feedback was present, learning motivation was significantly higher in the pedagogical agent condition than in the no pedagogical agent condition (*F* = 4.329, *p* = 0.040, *η*^2^ = 0.039). These results indicate that the combination of pedagogical agents and emotional feedback is conducive to enhancing learners’ motivation to learn.

### 4.4 Cognitive load

In terms of extraneous cognitive load, neither the pedagogical agent [*F*(1, 108) = 0.035, *p* = 0.852, *η*^2^ = 0.000] nor emotional feedback [*F*(1, 108) = 0.056, *p* = 0.813, *η*^2^ = 0.001] had significant main effects, but their interaction was marginally significant [*F*(1, 108) = 3.167, *p* = 0.078, *η*^2^ = 0.029]. Further simple effects analysis was conducted, as shown in Figure 8d. There was no significant difference between the emotional feedback and no emotional feedback conditions (*F* = 1.899, *p* = 0.171, *η*^2^ = 0.017). Similarly, when the pedagogical agent was present, there was no significant difference between the emotional feedback and no emotional feedback conditions (*F* = 1.157, *p* = 0.285, *η*^2^ = 0.011). When emotional feedback was absent, there was no significant difference between the pedagogical agent and no pedagogical agent conditions (*F* = 1.238, *p* = 0.268, *η*^2^ = 0.011). Likewise, when emotional feedback was present, there was no significant difference between the pedagogical agent and no pedagogical agent conditions (*F* = 1.968, *p* = 0.164, *η*^2^ = 0.018).

In terms of intrinsic cognitive load, neither the pedagogical agent (*F* = 1.690, *p* = 0.196, *η*^2^ = 0.016) nor emotional feedback [*F*(1, 108) = 0.067, *p* = 0.797, *η*^2^ = 0.001] had significant main effects, and there was no interaction effect between them [*F*(1, 108) = 1.429, *p* = 0.235, *η*^2^ = 0.013].

### 4.5 Academic emotions

For positive emotions, neither the pedagogical agent [*F*(1, 108) = 0.139, *p* = 0.710, *η*^2^ = 0.001] nor emotional feedback [*F*(1, 108) = 1.531, *p* = 0.219, *η*^2^ = 0.014] had significant main effects, and there was no interaction effect between them [*F*(1, 108) = 0.483, *p* = 0.489, *η*^2^ = 0.004].

For negative emotions, neither the pedagogical agent [*F*(1, 108) = 0.271, *p* = 0.604, *η*^2^ = 0.003] nor emotional feedback [*F*(1, 108) = 0.159, *p* = 0.691, *η*^2^ = 0.001] had significant main effects, and there was no interaction effect between them [*F*(1, 108) = 0.052, *p* = 0.820, *η*^2^ = 0.000].

### 4.6 Social presence

In terms of social presence, neither the pedagogical agent [*F*(1, 108) = 0.040, *p* = 0.842, *η*^2^ = 0.000] nor emotional feedback [*F*(1, 108) = 2.417, *p* = 0.123, *η*^2^ = 0.022] had significant main effects, and there was no significant interaction effect between them [*F*(1, 108) = 0.008, *p* = 0.928, *η*^2^ = 0.000].

### 4.7 Learning engagement

The main effect of the pedagogical agent on learning engagement was not significant [*F*(1, 108) = 2.160, *p* = 0.145, *η*^2^ = 0.020], whereas the main effect of emotional feedback was significant [*F*(1,108) = 4.459, *p* = 0.035, *η*^2^ = 0.041]. There was no interaction effect between the teaching agent and emotional feedback [*F*(1, 108) = 0.948, *p* = 0.332, *η*^2^ = 0.009].

## 5 Discussion

### 5.1 How does the presence of pedagogical agent impact learning performance in online formative assessment?

This study rejected H1a, which proposed that the presence of a feedback agent did not reduce learners’ negative emotions, nor did it increase their positive emotions, learning motivation, social presence, and learning engagement. Contrary to expectations, the study found that pedagogical agents reduced learners’ transfer scores but had no effect on retention scores. This outcome contradicts the predictions of Social Presence Theory and Social Agency Theory. One possible explanation is that, despite the use of social cues such as anthropomorphic images, gaze, and oral narration by the pedagogical agents in the study, these cues were not sufficient to trigger a sense of social presence in learners, and thus failed to produce the corresponding positive effects to enhance learners’ learning. Some studies have pointed out that the social presence generated by agents depends more on whether the agents provide enough social cues (Poinsot et al., 2022). Therefore, future research could consider further enhancing the interactivity and personalized expression of pedagogical agents to stimulate learners’ social presence and academic emotions more effectively. Situated in the context of online formative assessment, the pedagogical agent in this study is presented as a dynamic “peer” figure, frequently appearing at the critical moments when learners complete each task to provide feedback. This high-frequency, concrete social presence may also cause learners to disperse their cognitive resources on the “distracting” no-task features of the agent, such as facial expressions and eye movements, thereby interfering with information processing and affecting knowledge transfer. Therefore, when introducing pedagogical agents into formative assessment environments, their social presence should be carefully designed to minimize potential interference effects. There are two possible explanations for the non-significant impact of the agent on academic emotions and learning motivation. The first explanation is related to the interactive cues of the agents. Mayer (2020) suggests that interactive cues such as gestures and voice of pedagogical agents can influence motivation and learning. Although our animated agents featured head movements, lip-synced narration, and gaze, these cues might not have been sufficient to stimulate learners’ motivation or enhance their learning experience. The second explanation involves learner preferences. Learners tend to exhibit better situational motivation and learning experience when interacting with pedagogical agents that align with their preferences, as they have “expectations of use” for the agents (Domagk, 2010). The online learning system in this study used a uniform “peer Figure” as the feedback agent, which may not meet the preferences of all learners.

According to Cognitive Load Theory, incorporating pedagogical agents into the online learning environment may increase extraneous cognitive load, as learners are required to process additional (irrelevant) information. However, for H1b, we found that the agent factor had no effect on intrinsic or extraneous cognitive load but significantly affected learners’ study duration, which does not support Cognitive Load Theory. Therefore, H1b was not supported. This is consistent with the findings of Van der Meij et al. (2015) and Lin et al. (2013). Cognitive Load Theory indicates that extraneous cognitive load originates from the presentation of learning materials and interface design (Sweller et al., 2019). Some studies have shown that poorly designed pedagogical agents may increase learners’ cognitive load, especially extraneous cognitive load (Tao et al., 2022). However, this study did not find that the use of a pedagogical agent increased learners’ cognitive load. Therefore, we can conclude that the use of a pedagogical agent in this study did not generate additional interactive information and cues, and thus did not increase learners’ extraneous cognitive load. Meanwhile, the study found that the presence of a pedagogical agent does not affect learners’ intrinsic cognitive load, which is consistent with the findings of Ahuja et al. (2021). This may be because intrinsic cognitive load depends on the complexity of the task itself and the learners’ prior knowledge (Sweller et al., 2019), while a pedagogical agent, as an external element, has relatively limited influence on it. Furthermore, studies have shown that poorly designed pedagogical agents can increase learners’ cognitive load, especially extraneous cognitive load (Tao et al., 2022). However, this study did not find that the use of pedagogical agents increased learners’ cognitive load, so we can conclude that the pedagogical agent used in the study did not generate additional interactive information and cues, and thus did not increase learners’ cognitive load. However, the study duration of learners in the agent condition was significantly shorter than that of learners in the non-agent condition. A possible explanation is that under non-agent conditions, learners have more direct access to study materials, thereby spending more time and effort on the content itself.

### 5.2 How does emotional feedback impact learning performance in online formative assessment?

This study partially supports H2a, finding that emotional feedback has a significant positive impact on learning engagement. Self-Determination Theory (Deci and Ryan, 1985) suggests that individuals require external support for autonomy, competence, and relatedness when pursuing goals. Positive emotional feedback may fulfill these needs, such as by enhancing autonomy and competence through encouragement and recognition, prompting learners to engage more actively in learning activities. However, consistent with the findings of Horovitz and Mayer (2021), emotional feedback in the present study did not significantly influence academic emotions, learning motivation, social presence, or performance. Lang et al. (2022) argue that emotional feedback can elicit positive emotional responses in learners, thereby enhancing motivation and enriching the overall learning experience. Supporting this view, previous research has demonstrated that emotional feedback tends to improve learning outcomes by influencing learners’ emotional states and motivational (or approach-related) behaviors (Lawson et al., 2021). Similarly, emotional response theory posits that the primary function of emotional feedback is to regulate learners’ emotional states in order to facilitate engagement and learning (Liew et al., 2017). Therefore, the absence of significant improvements in learners’ emotional states, learning experiences, and academic performance observed in this study may be attributed to the limited salience or effectiveness of emotional processing during the online learning experience, which may have prevented emotional feedback from translating into meaningful learning gains. An alternative explanation is that emotional feedback may be more effective in promoting delayed testing outcomes—reflecting deeper learning—than in enhancing immediate test performance (Roediger and Karpicke, 2006). Regarding the design of emotional feedback, although it is phrased positively, it remains a “standardized” text or voice generated by the system. This formal expression undermines emotional resonance, leading learners to perceive it as a mechanical response rather than genuine care. As a result, it fails to effectively generate positive emotions and social presence. For example, fixed encouraging feedback from chatbots in online learning may be perceived by learners as programmed feedback, making it difficult to establish an effective emotional connection (Ortega-Ochoa et al., 2024). Contrary to H2b, emotional feedback did not reduce extraneous cognitive load. This may be because extraneous cognitive load stems from task complexity and the way information is presented, while intrinsic cognitive load depends on the complexity of the task itself and the learners’ prior knowledge. Emotional feedback, as a way of presenting non-instructional information, cannot directly change task complexity and information structure. Therefore, it is not sufficient to affect the cognitive processes in complex learning because it does not involve supportive information for cognitive processing (Kim et al., 2007). A meta-analysis study by Cai et al. (2023) highlighted potential moderating variables of feedback effectiveness, such as feedback timing and feedback type. Therefore, subsequent in-depth research on emotional feedback should more meticulously explore how these moderating variables interact with emotional feedback and how they jointly affect learners’ cognitive load and learning outcomes.

### 5.3 How do pedagogical agents and emotional feedback interact in online formative assessment to impact learning performance?

The study results indicate that when feedback agents and emotional feedback are combined, learners exhibit the highest levels of overall motivation, partially supporting H3. This is in line with similar findings from previous research (dos Santos Alencar and de Magalhães Netto, 2020; Wang et al., 2022). According to the emotional response theory (Horan et al., 2012), pedagogical agents with positive verbal and non-verbal emotional cues are more likely to elicit learners’ emotional motivation processing. Specifically, the positive verbal and non-verbal communication exhibited by agents may affect learners’ learning performance by influencing their emotional responses. Some studies have also pointed out that there may be a synergistic effect between the presence of pedagogical agents and emotional feedback (Lang et al., 2024; Wang Y. et al., 2023). Specifically, pedagogical agents provide concrete social cues, while emotional feedback enhances the responsiveness of the interaction. When these two elements are combined, they may simulate a more anthropomorphic and supportive learning environment, thereby meeting learners’ emotional and social needs and thus stimulating a higher level of learning motivation. The value of this anthropomorphic interactive experience has also been emphasized in recent research on affective computing and intelligent teaching systems (Zheng et al., 2024). However, when either of them exists alone, the intensity of the emotional cues or social interaction hints may not be sufficient to trigger learners’ learning motivation. This also suggests that in future research, when designing agent-based feedback, we need to comprehensively consider the social interaction capabilities of the agent and the dynamics of emotional feedback to better meet learners’ social and emotional needs. Unlike one-time summative feedback, the online formative feedback used in this study emphasizes feedback and adjustment during the process, and learners’ acceptance and emotional experience of the feedback directly affect the continuity and proactivity of learning. Therefore, the synergistic mechanism of agents and emotional feedback has greater potential in this context. However, aside from a significant increase in learning motivation, learners’ emotional perception and learning performance were not enhanced. A study by Beege et al. (2020) also found similar results. It is particularly noted that there is a marginal interactive effect of agents and emotional feedback on learners’ transfer scores, potentially reducing transfer performance. This may be because learners have positive emotions and high expectations for highly anthropomorphic pedagogical agents, expecting social communication and interaction from them. If emotional feedback is inconsistent with learners’ actual performance or expectations, it may not have a positive impact, similar to the Uncanny Valley theory (Mori et al., 2012), where overly human-like robots may elicit human aversion, affecting the desired outcomes. Furthermore, pedagogical agents with positive emotional feedback, which are unrelated to the learning content itself, may increase learners’ cognitive load from the perspective of interference theory, thereby reducing their transfer scores. However, the study found no significant difference in cognitive load levels between the agent and emotional feedback combination group and the control group, which may be because the cognitive load scale used in the study was a subjective assessment questionnaire, potentially leading to recall bias (De Jong, 2010). Therefore, more direct measurement methods will be beneficial for subsequent research.

## 6 Conclusion

This study, through a 2 (Pedagogical agent: present vs. absent) × 2 (Emotional feedback: present vs. absent) experimental design, thoroughly investigated the impact of different feedback forms on learners’ performance and experience during the formative assessment process in an online learning environment. The findings offer a new perspective on research concerning pedagogical agents and feedback forms, and provide a reference for the optimization and development of future online learning systems.

Firstly, the study found that pedagogical agents did not significantly enhance learning outcomes in online formative assessment systems and might even negatively affect knowledge transfer, aligning with interference theory, which suggests that we should carefully consider the design of pedagogical agents when designing feedback systems. Secondly, the study emphasizes the significant role of emotional feedback in promoting learning motivation. The experimental results show that, in the context of formative assessment, pedagogical agents with emotional support can significantly enhance learners’ motivation, a finding that aligns with the emotional response theory, indicating that emotional factors are indispensable in the online learning process. Furthermore, the study indicates that pedagogical agents may increase cognitive load in the absence of emotional feedback, while emotional feedback can alleviate this burden to some extent. Lastly, the study also demonstrates that providing positive emotional feedback to online learners can significantly increase their learning engagement. This result further highlights the value of emotional feedback in the formative assessment process and suggests that we should place greater emphasis on integrating emotional feedback when designing agent feedback.

Although this study holds significant value, there are several limitations and implications for the future research. The sample of this study is composed of undergraduate students from a university, and the relatively homogeneous sample structure may limit the generalizability of the research findings. Future research should further expand the sample coverage to include groups with different age ranges, academic backgrounds, and learning needs, in order to verify the applicability and differential effects of pedagogical agents and emotional feedback across different populations. The study did not take into account diverse learner characteristics. Different learners may employ different emotion regulation strategies, and thus may benefit differently from emotional feedback and pedagogical agents (Wang et al., 2022). Furthermore, according to the expertise reversal effect (Johnson et al., 2015), learners with different levels of knowledge and experience have varying needs for feedback agents. Therefore, it is essential to adopt different feedback agents based on learners’ varying levels of knowledge and experience. In addition, the study primarily relied on subjective measurement tools to assess learning motivation, emotional perception, etc., which may lead to biased results. Therefore, in future studies, a variety of assessment methods could be employed (e.g., combining subjective reports with objective measurements) to test the effectiveness of agent feedback. For instance, eye-tracking measurement indicators (such as fixation points in areas of interest, fixation duration, and number of fixations) could be used to determine whether emotional agents act as guides for directing attention or as distractors that divert attention, thereby gaining a deeper understanding of learners’ attention patterns toward emotional agents during the learning process. Moreover, the effectiveness of feedback is contingent upon the actual actions taken by learners (Dawson et al., 2024). Consequently, subjective measurement tools may not fully capture how learners actually apply feedback to improve their learning. Future research should employ a combination of assessment methods, including objective observations and analyses of the learners’ practice processes, to more accurately explore the intrinsic relationship between feedback and learning improvement.

## 7 Implications

This study offers critical insights for educators regarding the design of feedback mechanisms in online learning environments. First, pedagogical agents in online formative assessment systems did not significantly enhance learning outcomes and may even hinder knowledge transfer. This suggests that educators should exercise caution when designing pedagogical agents to prevent them from becoming a distraction in the learning process. Second, the study found that pedagogical agents with emotional feedback significantly boost learning motivation and reduce extraneous cognitive load, thereby enhancing learning engagement. Therefore, educators should integrate emotional feedback into online formative assessment environments to create a more humanized and supportive digital learning environment.

## Data Availability

The raw data supporting the conclusions of this article will be made available by the authors, without undue reservation.
